# Supplementary material for: Predicting visual function by interpreting a neuronal wiring diagram
Source: Nature. 2024 Oct 2;634(8032):113–23. doi: 10.1038/s41586-024-07953-5 (PMC11446822; doi:10.1038/s41586-024-07953-5)
Supplement: Supplementary file 6 — CRF and ERF predictions for individual TmY4 and TmY9 cells. Analogous to Supplementary Data 3, but for TmY target types. Shown are the top four monosynaptic pathways, the strongest pathway passing through each of the top ten intermediary types (ranking from Extended Data Fig. 7), and the trisynaptic pathway Tm1–TmY–Dm3–TmY (see the section entitled Prediction of spatial normalization). [file 41586_2024_7953_MOESM6_ESM.zip › DataS4/TmY9q/720575940638027342.pdf]

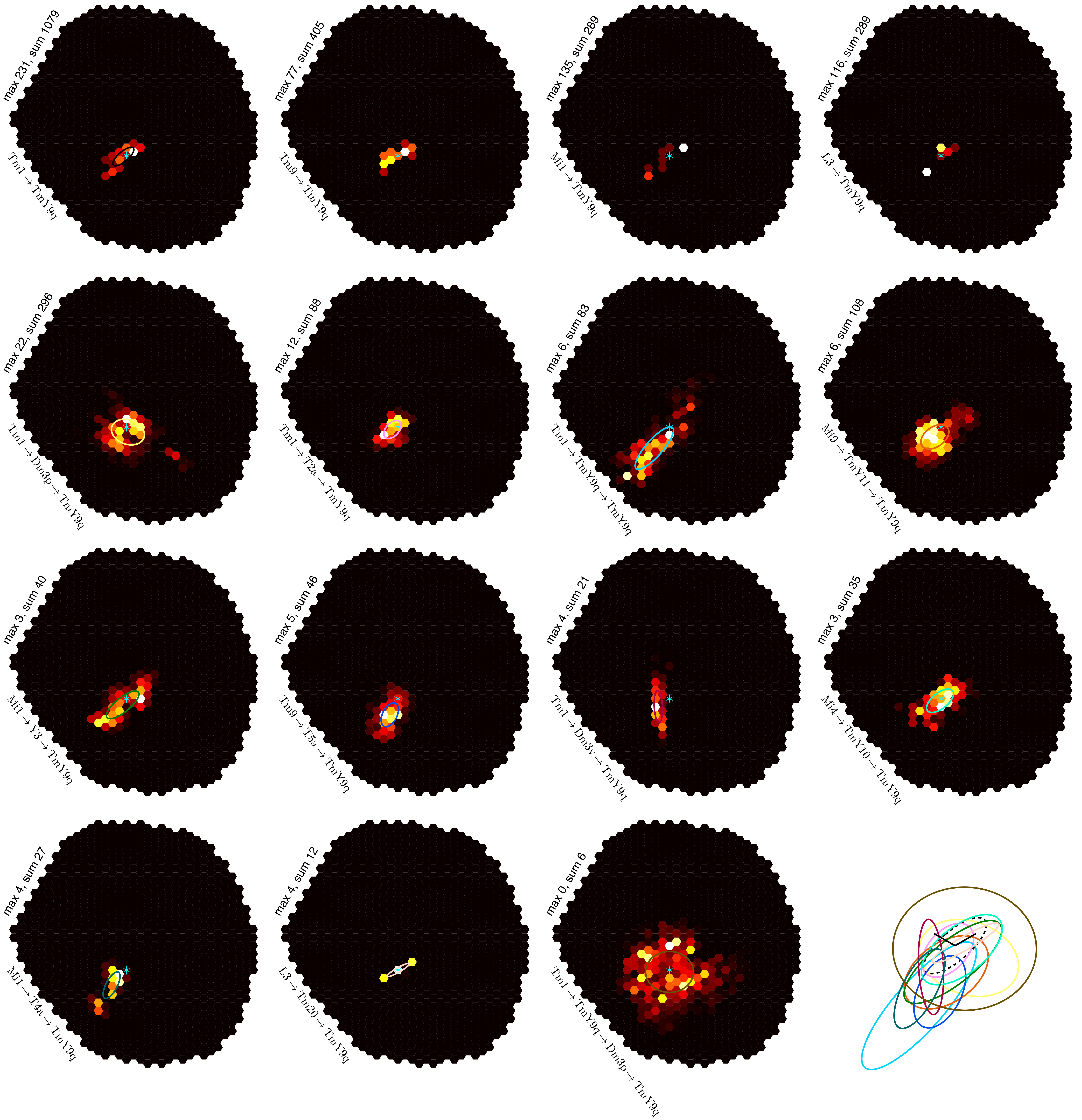

max 231, sum 1079
Tm1 → TmY9q
max 77, sum 405
Tm9 → TmY9q
max 135, sum 289
Mi1 → TmY9q
max 116, sum 289
L3 → TmY9q
max 22, sum 296
Tm1 → Dm3p → TmY9q
max 12, sum 88
Tm1 → T2a → TmY9q
max 6, sum 83
Tm1 → TmY9q → TmY9q
max 6, sum 108
Mi9 → TmY11 → TmY9q
max 3, sum 40
Mi1 → Y3 → TmY9q
max 5, sum 46
Tm9 → T5a → TmY9q
max 4, sum 21
Tm1 → Dm3v → TmY9q
max 3, sum 35
Mi4 → TmY10 → TmY9q
max 4, sum 27
Mi1 → T4a → TmY9q
max 4, sum 12
L3 → Tm20 → TmY9q
max 0, sum 6
Tm1 → TmY9q → Dm3p → TmY9q
